# Meckel’s Diverticulum Strangulation

**DOI:** 10.7759/cureus.14817

**Published:** 2021-05-03

**Authors:** Mohamed Ahmed, Mohamed Elkahly, Tito Gorski, Ahmed Mahmoud, Francis Essien

**Affiliations:** 1 Surgery, University of California, Riverside, USA; 2 Surgery, Riverside Community Hospital, Riverside, USA; 3 Surgery, Southwest Healthcare System, Murrieta, USA

**Keywords:** bowel obstruction, strangulation, lower abdominal pain, atypical appendicitis, volvulus, meckel’s diverticulum

## Abstract

Meckel’s diverticulum is the most common congenital anomaly of the small intestine. It is a true diverticulum containing all layers of the intestinal wall and results from the incomplete resolution of the omphalomesenteric duct. The majority of the cases are asymptomatic; however, diagnostic challenges arise when it becomes inflamed, causes gastrointestinal hemorrhage, intestinal obstruction, or when it protrudes through a potential abdominal opening (Littre’s hernia). We present a rare case of strangulated Meckel’s diverticulum as a result of axial torsion presenting with right lower quadrant abdominal pain.

## Introduction

Meckel’s diverticulum is the most common congenital anomaly of the gastrointestinal tract. It results from the persistence of the omphalomesenteric duct which connects the primitive gut to the yolk sac. Failure of obliteration of the duct (normally by the eighth week of gestation) may result in a variety of anatomic patterns, such as omphalomesenteric cysts, fistulae, and fibrous bands from the diverticulum to the umbilicus [1,2]. It follows the rule of twos, with 2% incidence, usually present before two years of age, two inches in length, two types of heterotopic mucosa, and two feet from the ileocecal valve [3]. The majority of Meckel’s diverticula are clinically silent, particularly in adults, and may be discovered incidentally during abdominal exploration or on diagnostic imaging. Symptomatic Meckel’s diverticulum may present with abdominal pain, symptoms of gastrointestinal bleeding, or bowel obstruction (secondary to intussusception or volvulus) [4]. Axial torsion with gangrene is very rare and has been scarcely reported [5,6].

## Case presentation

**Figure 1:**
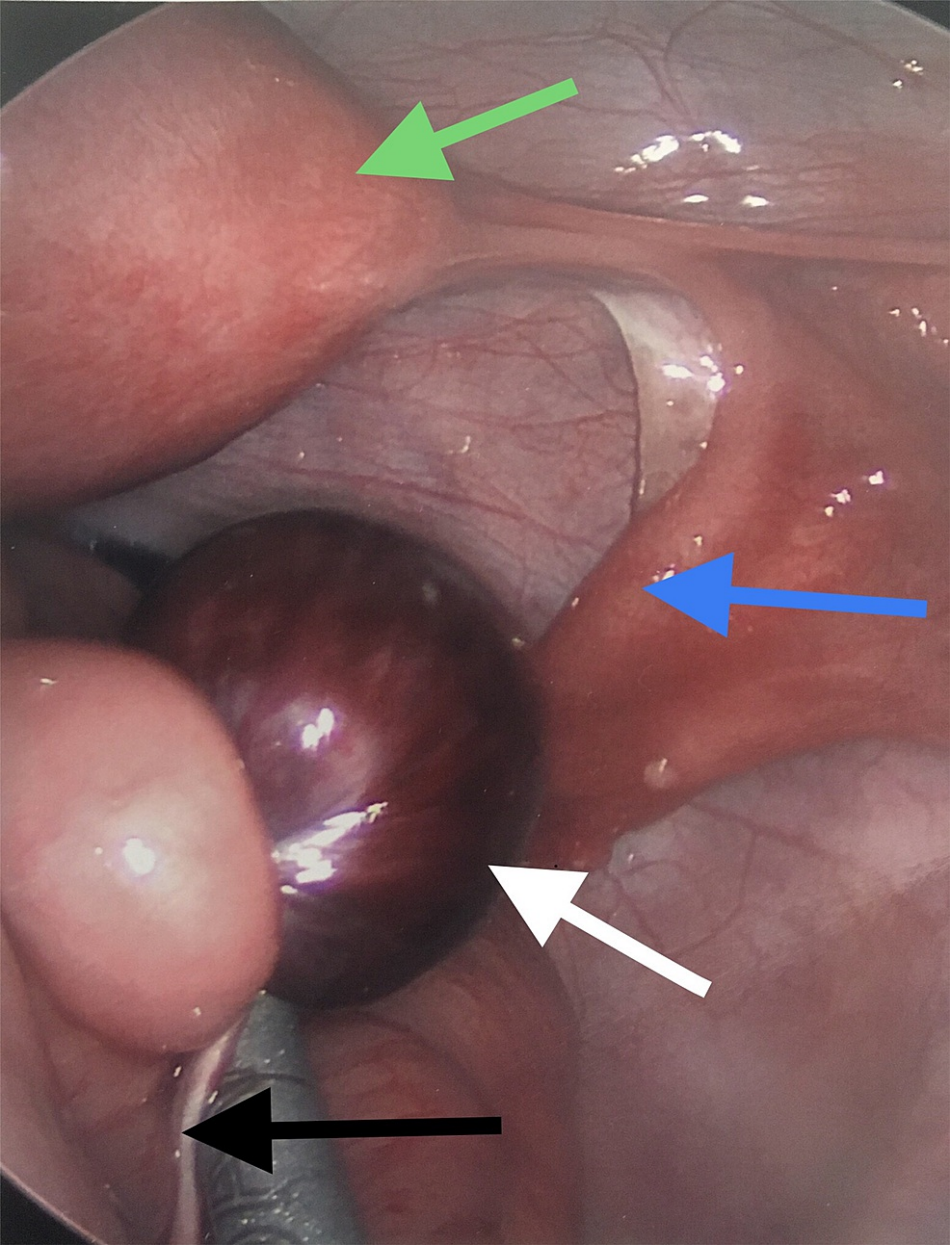
Diagnostic laparoscopy. Strangulated Meckel’s diverticulum (white arrow), a fibrous band (black arrow), uterus (green arrow), and Fallopian tube (blue arrow).

A 28-year-old female patient presented to our emergency room with a one-day history of right lower quadrant abdominal pain associated with nausea and low-grade fevers. Physical examination revealed right lower quadrant discomfort on deep palpation. Laboratory findings were normal except for a mildly elevated white blood cell count of 11.3 k/uL (normal range: 4.0-11.0 K/uL). Computed tomography revealed some fat stranding in the right lower quadrant; however, the appendix was not identified. The patient initially opted for non-operative management. After six hours of observation, the pain became severe and out of proportion to the physical examination findings. Diagnostic laparoscopy revealed a twisted small bowel, a gangrenous structure in the pelvis which was later identified as a strangulated Meckel’s diverticulum, and a fibrous band that extended to the anterior abdominal wall as the underlying cause (Figure 1).

**Figure 2:**
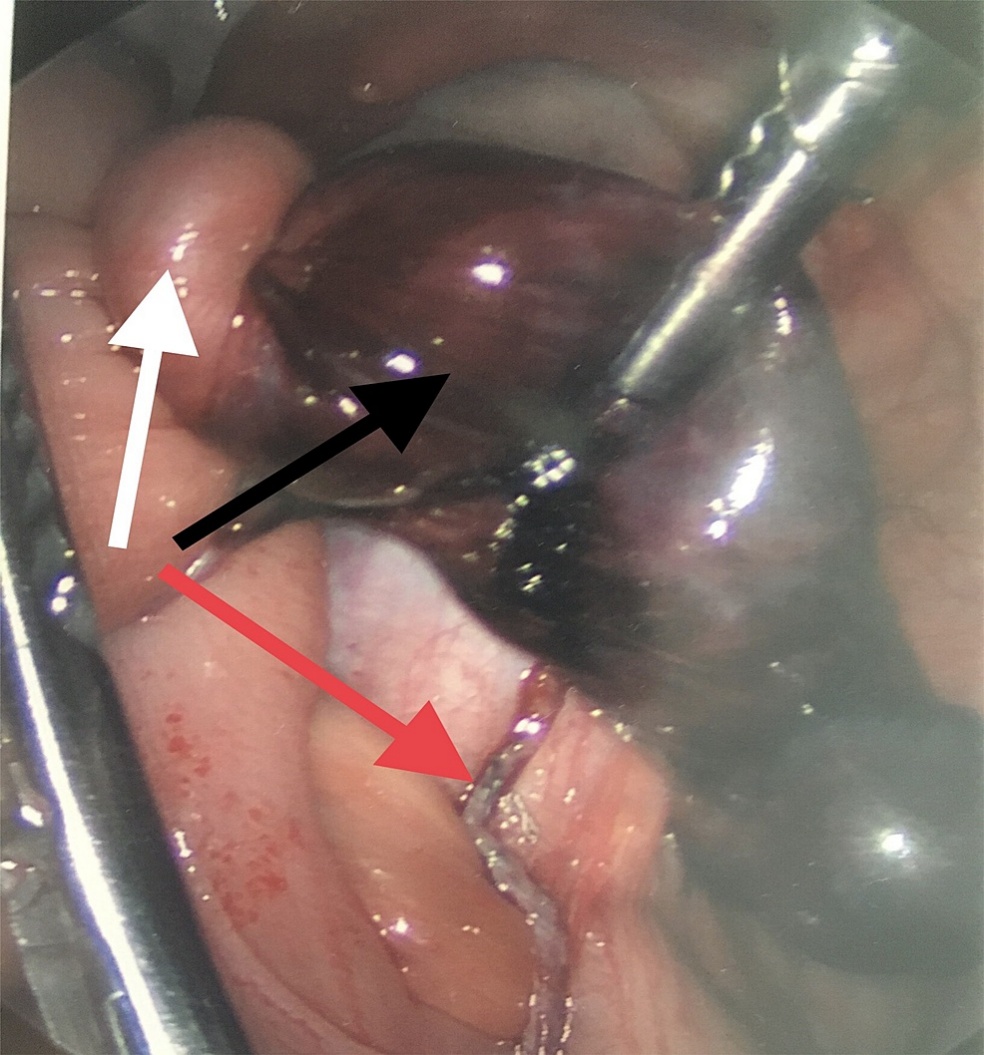
Band and appendix resected. Twisted small bowel (white arrow), gangrenous Meckel’s diverticulum (black arrow), and appendectomy site (red arrow).

Laparoscopic appendectomy, band excision, and reduction of the twisted terminal ileum was performed (Figure 2).

**Figure 3:**
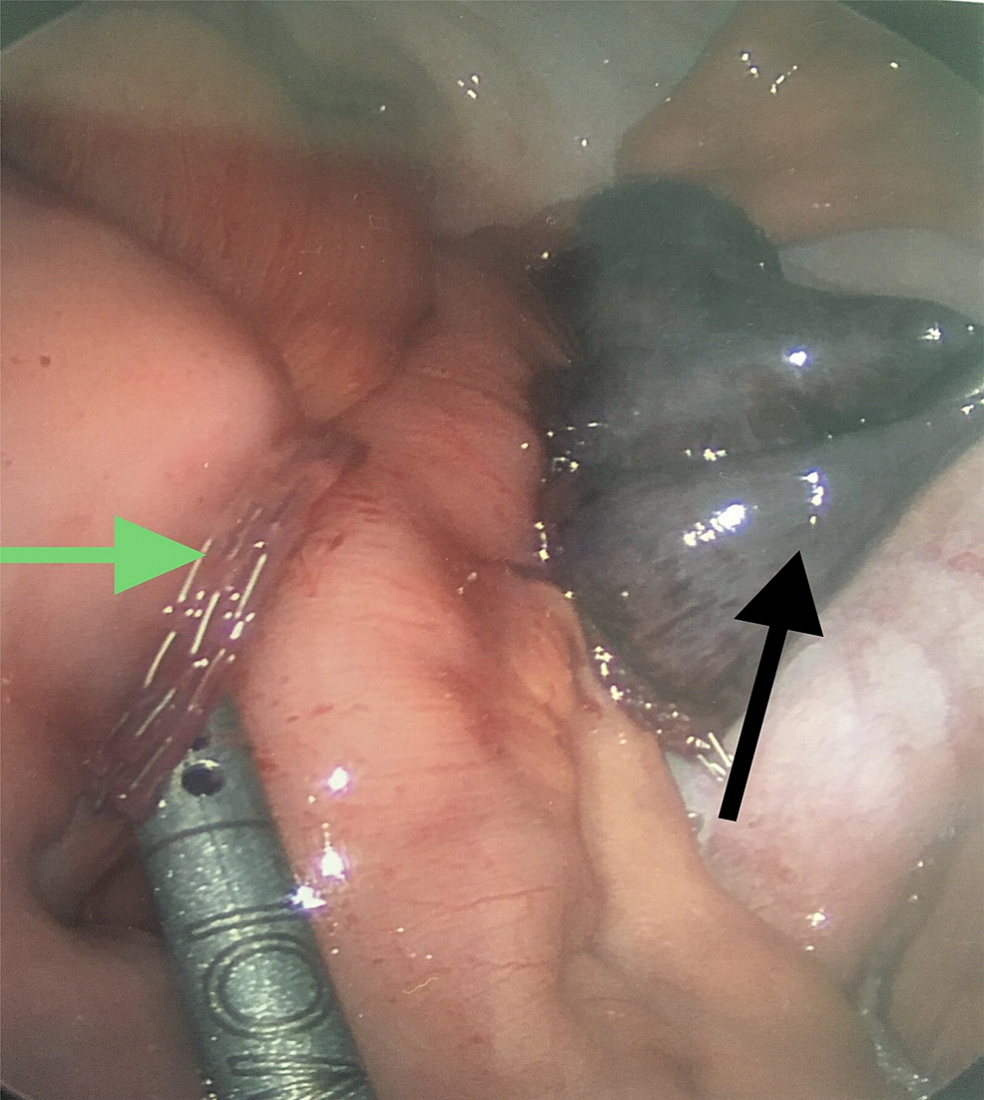
Meckel’s diverticulectomy. Transected gangrenous Meckel’s diverticulum (black arrow) and terminal ileum site of transection (green arrow).

Laparoscopic excision of the Meckel’s diverticulum was performed (Figure 3).

The patient did well and was discharged home on postoperative day two.

## Discussion

Meckel’s diverticulum is a true diverticulum containing all the layers of the gastrointestinal wall, and is the result of incomplete regression of the vitelline duct that connects the primitive gut to the yolk sac [7]. Patients are widely asymptomatic and symptoms are reported in 4%-7% of the cases. Ectopic gastric, pancreatic, duodenal, and colonic tissues can influence the management and outcome and are associated with symptomatic Meckel’s in general [8]. Complications in adults include bowel obstruction, diverticulitis, perforation, intussusception, rare malignancy, and vesico-diverticular fistulae. In the pediatric population, hemorrhage is seen in association with peptic ulceration as a result of ectopic gastric tissue.

Axial torsion is a rare complication due to the presence of a meso-diverticular band and having a narrow base creating an opening for bowel internal herniation [9,10]. Elective resection is not recommended in Meckel’s diverticulum found incidentally on imaging nor in asymptomatic patients >50 years of age when found during the course of an abdominal exploration [11]. Small bowel follow‐through, computed tomography including enterography, technetium 99 pertechnetate single-photon emission computed tomography scan, and resistance index scintigraphy can be used to show typical imaging features of Meckel’s diverticulum [12].

Meckel’s diverticula should be resected in all symptomatic patients, asymptomatic children when found incidentally during abdomen exploration, and in cases presenting with a concomitant meso-diverticular band to prevent future morbidities. Laparoscopic resection is feasible and safe [5].

## Conclusions

Symptomatic Meckel’s diverticula are often misdiagnosed as acute appendicitis and provide a diagnostic challenge. The management of Meckel’s diverticulum is controversial but includes resection in all symptomatic patients and asymptomatic children when found incidentally during the course of an abdominal exploration. Strangulation is rare and can be life-threatening. Diagnostic laparoscopy with laparoscopic resection is feasible and safe.
